# Intracellular hepatitis B virus increases hepatic cholesterol deposition in alcoholic fatty liver via hepatitis B core protein[S]

**DOI:** 10.1194/jlr.M079533

**Published:** 2017-11-13

**Authors:** Yaqi Wang, Ting Wu, Danqing Hu, Xinxin Weng, Xiaojing Wang, Pei-Jer Chen, Xiaoping Luo, Hongwu Wang, Qin Ning

**Affiliations:** Department and Institute of Infectious Disease* Hospital, Tongji Medical College, Huazhong University of Science and Technology, Wuhan 430030, China; Department of Pediatrics,§ Tongji Hospital, Tongji Medical College, Huazhong University of Science and Technology, Wuhan 430030, China; Graduate Institute of Clinical Medicine,† College of Medicine, National Taiwan University, Taipei 100, Taiwan

**Keywords:** alcoholic liver disease, hepatitis B virus, cholesterol

## Abstract

Hepatitis B virus (HBV) infection is a prevalent infectious disease with serious outcomes like chronic and acute hepatitis, cirrhosis, and hepatocellular carcinoma. However, the metabolic alteration by HBV is rarely taken into consideration. With the high prevalence of alcohol consumption and chronic HBV infection, their overlap is assumed to be an increasing latent hazard; although the extent has not been calculated. Moreover, the impact of chronic alcohol consumption combined with HBV on cholesterol metabolism is unknown. Six-week-old male FVB/Ncrl mice were hydrodynamically injected with a pGEM-4Z-1.3HBV vector and then fed an ethanol diet for 6 weeks. Serum biomarkers and liver histology, liver cholesterol levels, and cholesterol metabolism-related molecules were measured. In vitro assays with HBx, hepatitis B surface (HBs), or hepatitis B core (HBc) protein expression in HepG2 cells costimulated with ethanol were conducted to assess the cholesterol metabolism. HBV expression synergistically increased cholesterol deposition in the setting of alcoholic fatty liver. The increase of intrahepatic cholesterol was due to metabolic alteration in cholesterol metabolism, including increased cholesterol synthesis, decreased cholesterol degradation, and impaired cholesterol uptake. Overexpression of HBV component HBc, but not HBs or HBx, selectively promoted the hepatocellular cholesterol level.

Cholesterol homeostasis plays an important role in maintaining normal physiological functions, such as forming cell membranes, maintaining membrane integrity and fluidity, and supporting intracellular transport, cell signaling, and nerve conduction (1, 2). Apart from its importance for cell structure, cholesterol serves as a precursor for the biosynthesis of steroid hormones and bile acids as well (3). Dysregulated cholesterol homeostasis is a characteristic of numerous diseases, including atherosclerosis (4, 5), liver fibrosis (6), and even many cancers (7–9).

Hepatitis B virus (HBV) infection is a serious public health problem, with approximately 350–400 million individuals chronically infected worldwide who are at risk of developing liver cirrhosis and hepatocellular carcinoma (10). A recent study reports that HBV infection induces the expression of cholesterol synthesis genes. such as HMG-CoA reductase (HMGCR) and LDL receptor (LDLR) (11). Other research has observed that binding of HBV to the Na^+^ taurocholate cotransporting polypeptide (NTCP), which is a transporter for bile acid, limits its function, therefore promoting bile acid synthesis and cholesterol provision, resulting in the enhancement of cholesterol 7α-hydroxylase (CYP-7α) and compensatory upregulation of SREBP-2 and HMGCR (12).

Alcoholic liver disease (ALD) is also a major global burden, accounting for 0.9% of global mortality and 0.6% of disability-adjusted life-years in 2010 (13). As a major cause of chronic liver disease in Western countries, it contributes to 47.9% of liver cirrhosis-associated deaths in the USA (14). In the recent years, the production and consumption of alcoholic beverages have grown rapidly (15, 16) and ALD accounts for 14.8% of liver disease in Eastern countries (17). The spectrum of ALD includes alcoholic fatty liver disease, alcoholic hepatitis, and alcohol-related liver fibrosis and cirrhosis, among which alcoholic fatty liver is the earliest and most common subtype of ALD (18). Moreover, sporadic lines of evidence have demonstrated that chronic ethanol consumption disrupts cholesterol homeostasis (19). However, most of the patients suffering alcoholic fatty liver are asymptomatic, with few programs aimed at early detection of ALD (15).

In addition to the high prevalence of drinking alcohol in the general population, many alcoholics are also polysubstance abusers (20–22), and intravenous drug use is an important risk factor for HBV (23, 24). Thus, it is reasonable to suspect that a great portion of chronic hepatitis B (CHB) patients are suffering concomitant ALD. Currently, there is a lack of large scale epidemiological investigation of these comorbidities, and the study on the disease is limited. Previous reports showed that excessive alcohol consumption worsens the natural course of CHB. Heavy alcohol intake in CHB patients is associated with a higher risk for developing liver cirrhosis (25, 26), hepatocellular carcinoma (27–29), and liver-related mortality (30, 31). However, information on how ethanol consumption combined with HBV infection affects cholesterol metabolism in the liver is limited and elicits questions on: *1*) the metabolic consequences of ethanol consumption in the case of HBV infection; and *2*) how ethanol consumption associates with HBV persistence to affect metabolism in mice. In this project, we employed a mouse model of chronic alcohol consumption along with HBV expression to investigate the cholesterol metabolism associated with high risk of metabolic disorders.

## MATERIALS AND METHODS

### Plasmids

The pGEM-4Z-1.3HBV plasmid that contains a 1.3-fold over-length of the genotype B HBV genome was obtained from Pei-Jer Chen (Graduate Institute of Clinical Medicine, College of Medicine, National Taiwan University, Taipei, Tanwan).

The coding sequences of HBx, hepatitis B surface (HBs), and hepatitis B core (HBc) were amplified by PCR from the pGEM-4Z-1.3HBV plasmid. HBx, HBs, and HBc genes were amplified and cloned into the pCDH-CMV-MCS-EF1-copGFP vector (System Biosciences, Palo Alto, CA) with *Eco*RI/*Not*I, *Eco*RI /*Bam*HI, or *Xba*lI/*Eco*RI, respectively. The primers are listed in supplemental Table S1.

### Construction of animal models and animal experiments

Male FVB/Ncrl mice, 6–8 weeks of age, obtained from Vital River Laboratory Animal Technology (Beijing, China), were housed in a specific pathogen-free room. Ten micrograms of the pGEM-4Z-1.3HBV plasmid DNA or pGEM-4Z with random sequence plasmid DNA in a volume of phosphate-buffered saline equivalent to 8% of the mouse body weight was introduced by tail vein injection in 6–8 s (32). The mice were then randomly divided into four groups with seven to nine mice per group and were fed either a Lieber-DeCarli liquid ethanol diet or an isocaloric control diet (33). In brief, mice were gradually introduced to the ethanol-containing liquid diet as follows: liquid diet containing 1% ethanol (w/v) for 3 days, 3% for 4 days, and finally 5% for 6 weeks. Then, animals were euthanized and blood was collected. All animal procedures were performed according to the criteria outlined in the *Guide for the Care and Use of Laboratory Animals* (National Institutes of Health, Bethesda, MD) and with approval of the Animal Care and Use Committee of Huazhong University of Science and Technology.

### Serum hepatitis Be antigen and biochemical assays

The serum level of hepatitis Be antigen (HBeAg) was determined with an automated immunity analyzer (Architect i4000SR; Abbott Diagnostics, Abbott Park, IL) using the system reagent. Biomarkers that indicate the liver function were measured by an automated clinical chemistry analyzer (Roche Cobas 8000; Basel, Switzerland), including serum alanine aminotransferase (ALT) activities, aspartate aminotransferase (AST) activities, total bilirubin, direct bilirubin, alkaline phosphatase, and γ-glutathione. Serum lipid biomarkers, including serum triglyceride (TG), serum cholesterol, HDL, and LDL levels, were measured by the Cobas 8000 analyzer (Roche Cobas 8000) with the system reagents as well.

### Liver histopathological examination

Tissues were collected from mice at the indicated time points and 5 μm-thick sections of formalin-fixed and paraffin-embedded liver were processed for hematoxylin and eosin staining. Ten micrometer-thick sections of frozen liver tissue stained with Oil Red O (Sigma-Aldrich, St. Louis, MO) were examined to estimate the degree of hepatic steatosis.

### Liver cholesterol measurements

Liver tissues were homogenized and extracted with iced ethanol. A total cholesterol assay kit (Nanjing Jiancheng Bioengineering Institute, Nanjing, China) was used to measure liver cholesterol according to the manufacturer’s instructions.

### Cell culture, transfection, and alcohol stimulation

HepG2 cells were obtained from the American Type Culture Collection and cultured in Dulbecco’s modified Eagle’s medium containing 10% fetal bovine serum and 1% (v/v) penicillin-streptomycin at 37°C in a humidified atmosphere containing 5% CO_2_. Transfection was performed in 6-well plates containing 5 × 10^5^ cells/well using Lipofectamine 3000 (Invitrogen, Carlsbad, CA) according to the manufacturer’s instructions. Twenty-four hours after transfection, HepG2 cells were treated with 100 mM ethanol (34) in a closed modular incubator chamber (Billups-Rothenberg, San Diego, CA). At 48 h after transfection, cells were harvested for further analysis.

### RNA isolation and quantitative real-time PCR

Total RNA was extracted using Trizol reagent (Invitrogen) according to the manufacturer’s protocol. A reverse transcription reaction was performed with 2.0 μg of total RNA using ReverTra Ace qPCR RT kit (Toyobo, Osaka, Japan). SYBR Green Real-Time PCR Master Mix (Toyobo) was used for quantitative (q)RT-PCR in a final volume of 20 μl on a CFX96 RT-PCR system (Bio-Rad, Hercules, CA). Conditions were 95°C for 10 min followed by 40 cycles of amplification (95°C for 15 s, 58°C for 15 s, 72°C for 15 s). Following amplification, a melting curve analysis from 65°C to 95°C and continuous fluorescence was made. The average Ct (threshold cycle) of fluorescence units was applied to analyze the mRNA levels, which were normalized to a housekeeping gene (β-actin or GAPDH). The relative level of each gene was calculated using the 2^−ΔΔCt^ method. Primers are shown in supplemental Table S2.

### Protein extraction and Western blotting

A NE-PER nuclear and cytoplasmic extraction kit (Thermo Scientific, Rockford, IL) was used to extract nuclear and cytoplasmic proteins according to the manufacturer’s protocol. Each 20 μg protein sample was separated on an 8/10% sodium dodecyl sulfate-polyacrylamide gel. The proteins were then transferred onto polyvinylidene difluoride membranes (EMD Millipore, Darmstadt, Germany) using the Trans-Blot Turbo™ blotting system (Bio-Rad). After transferring, membranes were blocked with 5% BSA at room temperature for 2–3 h. The membranes were then incubated overnight at 4°C with anti-SREBP-2 (1:600; Abcam, Cambridge, UK), anti-HMGCR (1:5,000; Abcam), anti-LDLR (1:500; Abcam), anti-proprotein convertase subtilisin/kexin type 9 (PCSK9) (1:600; Proteintech, Wuhan, China), anti- CYP-7α (1:1,000; Santa Cruz Biotechnology, Santa Cruz, CA), anti-HBsAg (1:1,000; Abcam), anti-HBV xAg (1:200; Santa Cruz Biotechnology), anti-Hep B cAg (1:200; Santa Cruz Biotechnology), anti-GAPDH (1:10,000; Abcam), or anti-lamin B (1:300; Boster, Wuhan, China) primary antibodies, respectively. After washing with TBST, the membranes were incubated with horseradish peroxidase-linked secondary antibodies (1:5,000; Boster). An enhanced chemiluminescence Western blotting substrate kit (BioVision, SanFrancisco, CA) was used to detect specific proteins.

### Immunohistochemistry

The immunohistochemistry (IHC) assay was performed to determine expression patterns and subcellular locations of potential candidate markers in liver tissues. Five micrometer-thick sections of formalin-fixed and paraffin-embedded liver tissue were prepared. After dewaxing, antigen retrieval, and blocking with 3% goat serum, liver sections were stained with mouse anti-HBc antigen (HBcAg) (1:200 dilution; Abcam), rabbit anti-SREBP-2 (1:100 dilution; Proteintech) antibodies, or rabbit anti-PCSK9 (1:200 dilution; Proteintech) overnight at 4°C. Tissues were incubated with a horseradish peroxidase-linked secondary antibody (ZSBG-Bio; Beijing, China) and 3,3′-diaminobenzidine (ZSBG-Bio) following the manufacturer’s instructions. Liver sections were counterstained with hematoxylin.

### Immunofluorescence and immunofluorescence laser confocal microscopy assay

HepG2 cells were cultured on coverslips and treated as described above. At the indicated time points, cells were fixed with 4% paraformaldehyde and permeabilized with 0.5% Triton X-100 at room temperature for 20 min. Cells were then incubated with 3% normal goat serum for 1 h and stained with rabbit-anti SREBP-2 (1:50 dilution; Santa Cruz Biotechnology) overnight at 4°C. After washing with phosphate-buffered saline, cells were incubated in the dark with the secondary goat anti-rabbit antibody for 1 h at 37°C. Nuclei were stained with 4′,6-diamidino-2-phenylindole (Santa Cruz Biotechnology) for 5 min. After mounting, the samples were analyzed by fluorescence microscopy or laser confocal microscopy.

### Statistical analyses

Data are expressed as mean ± SEM. Statistical analyses were performed using a one-way ANOVA and analyzed further by Newman-Keuls test (for Gaussian distribution data) or Kruskal-Wallis test (as a nonparametric test) for statistical significance. Differences among groups were considered to be statistically significant at *P* < 0.05. Statistical analyses were performed using GraphPad Prism 5 software (GraphPad, La Jolla, CA).

## RESULTS

### Hydrodynamic injection and excessive ethanol intake lead to HBV persistence with liver steatosis in FVB/N mice

**Fig. 1.**
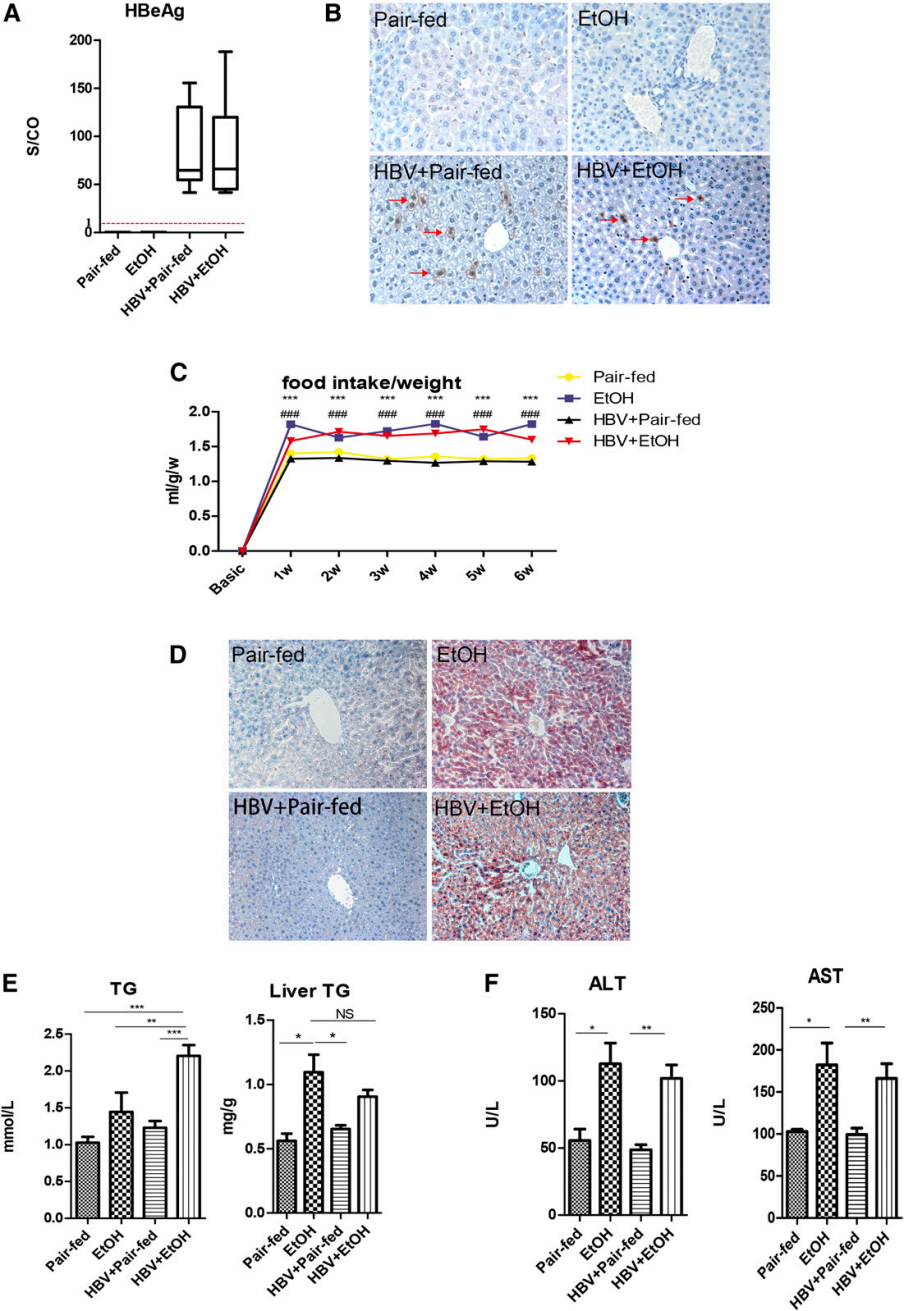
Hydrodynamic injection with pGEM-4Z1.3HBV and chronic alcohol feeding lead to copresence of HBV persistence and liver steatosis in FVB/N mice. A: Serum HBeAg levels. The levels of HBeAg in sera are shown as signal-to-control ratio (S/CO). The dotted red line represents the cut-off value of HBeAg. B: Liver HBcAg expression by immunohistochemical staining (red arrows; original magnification, ×400). C: Food intake/weights [***P < 0.001, pair-fed vs. ethanol (EtOH); ^###^*P* < 0.001, HBV + pair-fed vs. HBV + EtOH]. D: Oil Red O staining (original magnification, ×200). E: Serum TG level and liver TG level. F: Serum ALT and AST levels (n_1_ = 7, n_2_ = 9, n_3_ = 7, n_4_ = 8; n_1_, pair-fed; n_2_, EtOH; n_3_, HBV + pair-fed; n_4_, HBV + EtOH). Data are mean ± SEM. *P < 0.05; **P < 0.01; ***P < 0.001.

As HBV is a host-restricted virus, we employed tail vein injection of plasmid containing 1.3-fold HBV genome to achieve the persistence of HBV in murine hepatocytes, as described previously, followed by an alcohol-containing diet (32). As the results show, HBeAg was detectable in the serum, suggesting the persistence of HBV in the whole course of the experiments (**Fig. 1A**).

To ascertain the persistence of HBV in FVB/N mice (HBV mice), IHC staining was performed on the livers. It is noted that cytoplasmic HBcAg is an indicator of HBV replication (35), whereas nuclear HBcAg is stable even in the absence of HBV replication (36). IHC staining of the liver sections showed that HBcAg was expressed persistently in the cytoplasm of hepatocytes in the HBV groups (Fig. 1B). Collectively, these results demonstrate that the HBV DNA delivery endowed HBV persistence in the mouse liver.

Chronic alcohol consumption induced alcoholic fatty liver. Each mouse consumed an equal volume of liquid diet with or without ethanol intake per day. As shown in Fig. 1C, we observed significantly increased food intake/weights in the ethanol treatment groups; while an obvious decrease of food intake/weights in pair-fed groups during the 6 week dietary intervention suggested that mice treated with ethanol diet were suffering from alcohol consumption. Liver pathology using Oil Red O staining showed that, after 6 weeks, small lipid droplets appeared in the hepatocytes of ethanol-fed mice, but pair-fed mice exhibited normal hepatocytes (Fig. 1D). There was no significant difference of steatosis observed between the HBV group with ethanol feeding and ethanol-fed alone mice without HBV persistence in the liver, but the serum TG level was evidently increased in the HBV group with ethanol feeding (Fig. 1E). Serum ALT and AST levels were slightly elevated in the ethanol-fed groups (*P* < 0.05) (Fig. 1F). Total bilirubin and direct bilirubin were undetectable in the pair-fed groups, while the levels were weakly increased in the ethanol-fed mice (supplemental Table S3). These results indicated that there was mild inflammation in the liver of mice, but it was mainly caused by ethanol administration.

### Chronic ethanol consumption combined with HBV further increases cholesterol in vivo

**Fig. 2.**
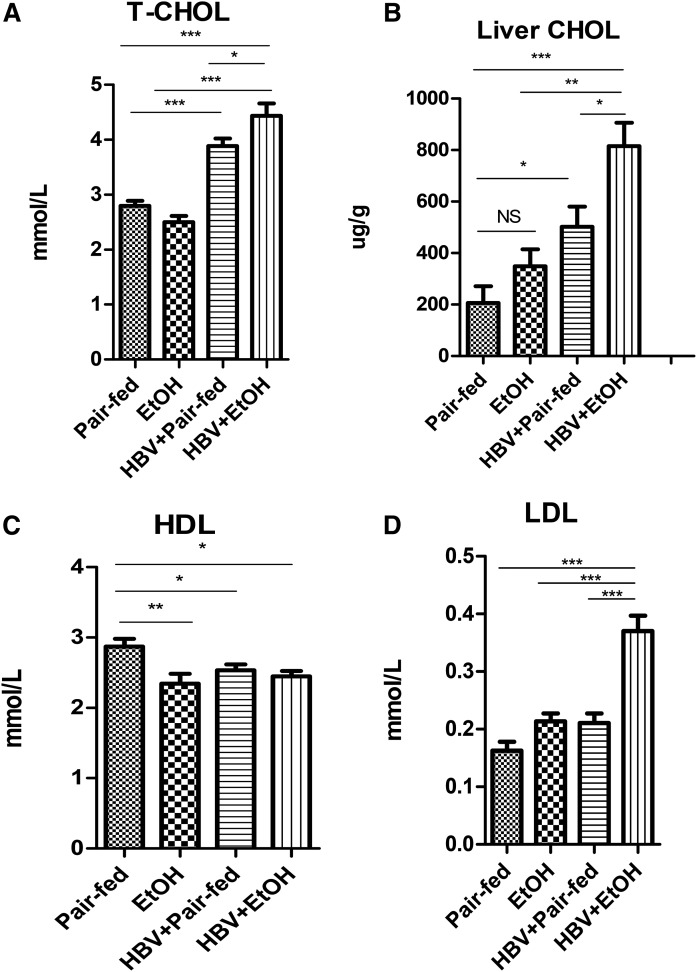
Chronic alcohol feeding combined with HBV persistence induced increases of serum and hepatic total cholesterol levels. A: Serum total cholesterol levels (T-CHOL). B: Liver total cholesterol levels (Liver Chol). C: Serum HDL levels. D: Serum LDL levels [n_1_ = 7, n_2_ = 9, n_3_ = 7, n_4_ = 8; n_1_, pair-fed; n_2_, ethanol (EtOH); n_3_, HBV + pair-fed; n_4_, HBV + EtOH]. Data are mean ± SEM. *P < 0.05; **P < 0.01; ***P < 0.001.

Interestingly, HBV delivery alone induced an increase in cholesterol level in the serum and liver compared with the pair-fed control mice (*P* < 0.001) (**Fig. 2A, B**). And ethanol consumption combined with HBV further synergically elevated the total cholesterol level in the serum and liver significantly compared with the other three groups [pair-fed HBV alone (*P* < 0.05), ethanol-fed alone (*P* < 0.001), and pair-fed control (*P* < 0.001)]. In contrast, the serum HDL level decreased in the HBV group treated with ethanol (Fig. 2C), while the LDL level significantly increased compared with the other groups (*P* < 0.001) (Fig. 2D).

### Chronic ethanol consumption combined with HBV synergistically promotes the cholesterol biosynthesis pathway

**Fig. 3.**
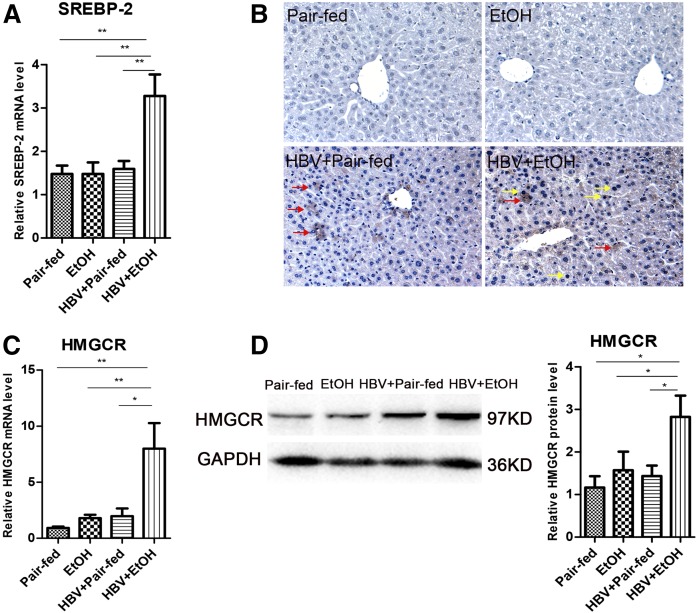
Chronic alcohol exposure combined with HBV persistence-induced increases in hepatic total cholesterol levels were associated with enhanced cholesterol biosynthesis pathway in FVB mice. A: Gene expression analysis of SREBP-2 by real-time PCR in the liver of FVB mice [n_1_ = 7, n_2_ = 9, n_3_ = 7, n_4_ = 8; n_1_, pair-fed; n_2_, ethanol (EtOH); n_3_, HBV + pair-fed; n_4_, HBV + EtOH]. B: Expression of SREBP-2 in the hepatocytes by IHC (red arrows, cytoplasmic SREBP-2; yellow arrows, nuclear SREBP-2; original magnification, ×400). C: Gene expression analysis of HMGCR by real-time PCR (n_1_ = 7, n_2_ = 9, n_3_ = 7, n_4_ = 8; n_1_, pair-fed; n_2_, EtOH; n_3_, HBV + pair-fed; n_4_, HBV + EtOH). D: Representative Western blot and relative quantitation of HMGCR protein levels in FVB mice. GAPDH was used as a loading control. Data are mean ± SEM. *P < 0.05; **P < 0.01; ***P < 0.001.

These increased cholesterol levels in the serum and liver indicated that ethanol administration combined with HBV may disrupt hepatic cholesterol metabolism. Cholesterol biosynthesis is essentially regulated by transcription factor SREBP-2 in hepatocytes. Thus, we measured the expression of SREBP-2 both in mRNA and in the nucleoprotein level in the liver. As shown in **Fig. 3A**, the mRNA level of SREBP-2 was upregulated significantly in HBV mice treated with ethanol compared with pair-fed mice (*P* < 0.01), ethanol-fed mice (*P* < 0.01), and HBV mice (*P* < 0.01). The activation of SREBP-2 was determined by detecting its nuclear translocation. More active nuclear translocation was observed in nuclei of HBV mice fed ethanol (Fig. 3B). This suggested that chronic ethanol exposure combined with HBV persistence enhanced de novo cholesterol synthesis in the liver.

Then, we examined the effects of ethanol combined with HBV on the expression of HMGCR, the rate-limiting enzyme involved in cholesterol synthesis, mediated by SREBP-2. The mRNA level of HMGCR was increased 4- to 8-fold in alcohol-consuming HBV mice compared with pair-fed mice (*P* < 0.01), ethanol-fed mice (*P* < 0.01), and HBV mice (*P* < 0.05) (Fig. 3C). The expression of HMGCR protein was also obviously increased in HBV mice that were fed with ethanol (Fig. 3D).

### Chronic alcohol consumption combined with HBV synergistically inhibits the cholesterol uptake and degradation pathway in vivo

**Fig. 4.**
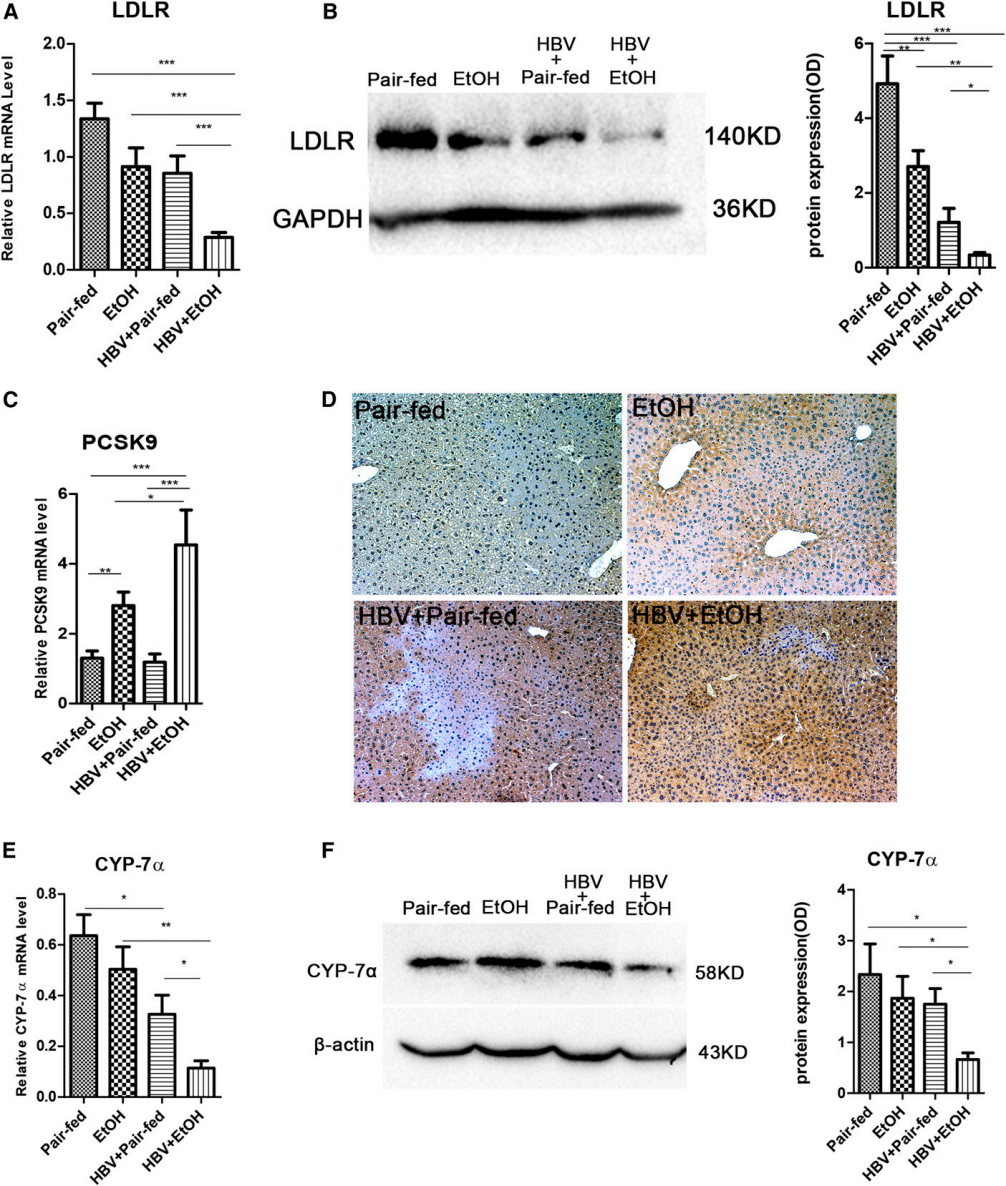
Chronic alcohol consumption combined with HBV persistence reduced hepatic LDLR levels via PCSK9 upregulation and inhibited the expression of CYP-7α in FVB mice. A: Gene expression analysis of LDLR by real-time PCR (n_1_ = 7, n_2_ = 9, n_3_ = 7, n_4_ = 8; n_1_, pair-fed; n_2_, ethanol (EtOH); n_3_, HBV + pair-fed; n_4_, HBV + EtOH). B: Representative Western blots and relative quantitation of LDLR proteins in the liver. C: Gene expression analysis of PCSK9 by real-time PCR (n_1_ = 7, n_2_ = 9, n_3_ = 7, n_4_ = 8; n_1_, pair-fed; n_2_, EtOH; n_3_, HBV + pair-fed; n_4_, HBV + EtOH). D: Expression of PCSK9 in the hepatocytes by IHC (original magnification, ×200). E: Gene expression analysis of CYP-7α by real-time PCR in FVB mice (n_1_ = 7, n_2_ = 9, n_3_ = 7, n_4_ = 8; n_1_, pair-fed; n_2_, EtOH; n_3_, HBV + pair-fed; n_4_, HBV + EtOH). F: Representative Western blots and relative quantitation of CYP-7α protein levels in FVB mice. GAPDH/β-actin was used as a loading control. Data are mean ± SEM. *P < 0.05; **P < 0.01; ***P < 0.001.

LDL is taken up via the LDLR and then de-esterified in late endosomes. Cholesterol esters are hydrolyzed into free cholesterol and fatty acids, and free cholesterol returns to the plasma membrane or is used for cellular needs (37). In our study, the expression of LDLR was decreased 3- to 5-fold in ethanol-fed mice with HBV compared with pair-fed mice (*P* < 0.001), ethanol-fed mice (*P* < 0.001), and HBV-persistence mice (*P* < 0.001) (**Fig. 4A**). LDLR protein levels were also decreased obviously in the combined treatment group (Fig. 4B). For further study of its upstream regulation, we investigated the expression of PCSK9, which promotes degradation of LDLR to increase plasma cholesterol levels (38). Our data showed that the hepatic gene and protein expression of PCSK9 were elevated significantly in ethanol-fed mice with HBV compared with pair-fed, ethanol-fed, and HBV-persistence mice (Fig. 4C, D). Consistent with this, the serum cholesterol level was increased significantly compared with the other three groups (Fig. 2A).

An important degradation pathway of cholesterol involves its conversion into bile acids. CYP-7α is the rate-limiting enzyme of this pathway. Therefore, we analyzed the expression of CYP-7α in the liver. The results showed that, in alcohol consumption combined with HBV mice, CYP-7α mRNA expression was decreased 3- to 6-fold compared with pair-fed mice (*P* < 0.001), ethanol-fed mice (*P* < 0.01), and HBV mice (*P* < 0.05) (Fig. 4D). The CYP-7α protein level was also downregulated in alcohol consumption combined with HBV mice (Fig. 4E). Overall, our data indicated that the combination of ethanol and HBV has a synergistic effect to inhibit the uptake and degradation of cholesterol.

### Chronic alcohol exposure combined with HBV synergistically disrupts cholesterol metabolism pathways in vitro

**Fig. 5.**
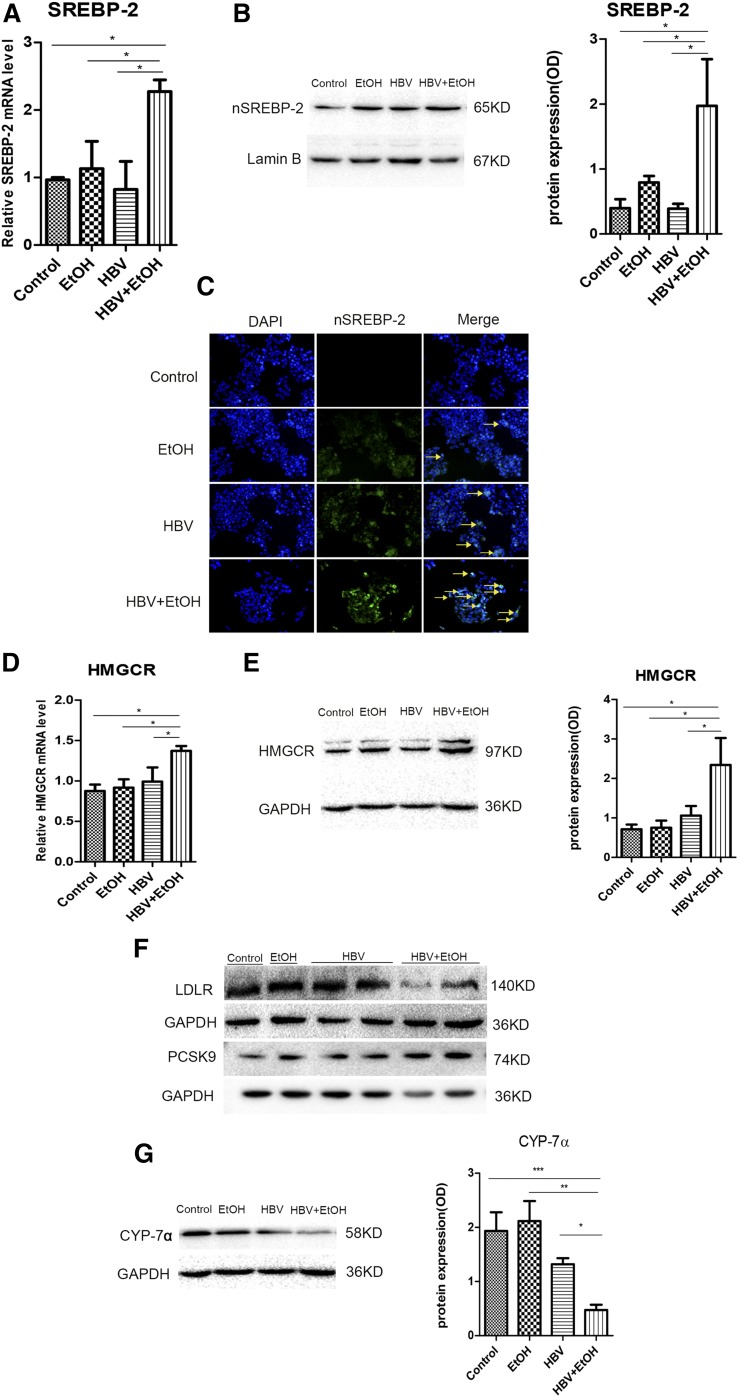
Chronic alcohol exposure combined with HBV persistence influenced the cholesterol metabolism pathway in vitro. A: Gene expression analysis of SREBP-2 by real-time PCR in HepG2 cells. B: Representative Western blots and relative quantitation of nuclear SREBP-2 protein levels in HepG2 cells. Lamin B was used as a loading control. C: Expression of nuclear SREBP-2 (nSREBP-2) in HepG2 cells by immunofluorescence (yellow arrows: original magnification, ×200). D: Gene expression analysis of HMGCR by real-time PCR in HepG2 cells. E: Representative Western blots and relative quantitation of HMGCR protein levels in HepG2 cells. F: Representative Western blots of LDLR and PCSK9 protein levels in HepG2 cell lines. G: Representative Western blots and relative quantitation of CYP-7α protein levels in HepG2 cell lines. GAPDH was used as a loading control. Data are mean ± SEM of three separate experiments done in triplicate. *P < 0.05; **P < 0.01; ***P < 0.001.

To further investigate the combined effects of ethanol and HBV on cholesterol metabolism, we mimicked ethanol stimulation in cooperation with HBV persistence in vitro by using HepG2 cells. The data shown in **Fig. 5A, B** suggested that the mRNA level and the mature form of SREBP-2 protein were significantly induced in HepG2 cells in response to ethanol stimulation combined with HBV (*P* < 0.05) compared with other groups. Consistent with these results, the nuclear SREBP-2 was obviously enhanced in the ethanol stimulation combined with HBV group in HepG2 cells (Fig. 5C). Furthermore, the expression of HMGCR mRNA and protein were also significantly upregulated in the combined treatment group (*P* < 0.05) compared with other groups (Fig. 5D, E). In addition, LDLR, a key receptor on hepatocytes for cholesterol intake, was decreased significantly in ethanol stimulation combined with intracellular HBV compared with the other three groups (Fig. 5F). Consistently, PCSK9, as an important enzyme responsible for LDLR degradation, was increased significantly (Fig. 5F). As an important rate-limiting enzyme for cholesterol degradation, a significant decrease of CYP-7α protein levels was observed in HepG2 cells transfected with HBV and treated with ethanol in vitro (Fig. 5G). Similar results were also obtained in the Huh7 cell line (see supplemental Fig. S1). Collectively, these results further suggested that excessive ethanol consumption and HBV persistence result in synergistic cholesterol accumulation as demonstrated by increased cholesterol synthesis and decreased intake and degradation.

### Overexpression of HBc protein combined with ethanol stimulation increases the cholesterol biosynthesis pathway and inhibits the cholesterol degradation pathway

**Fig. 6.**
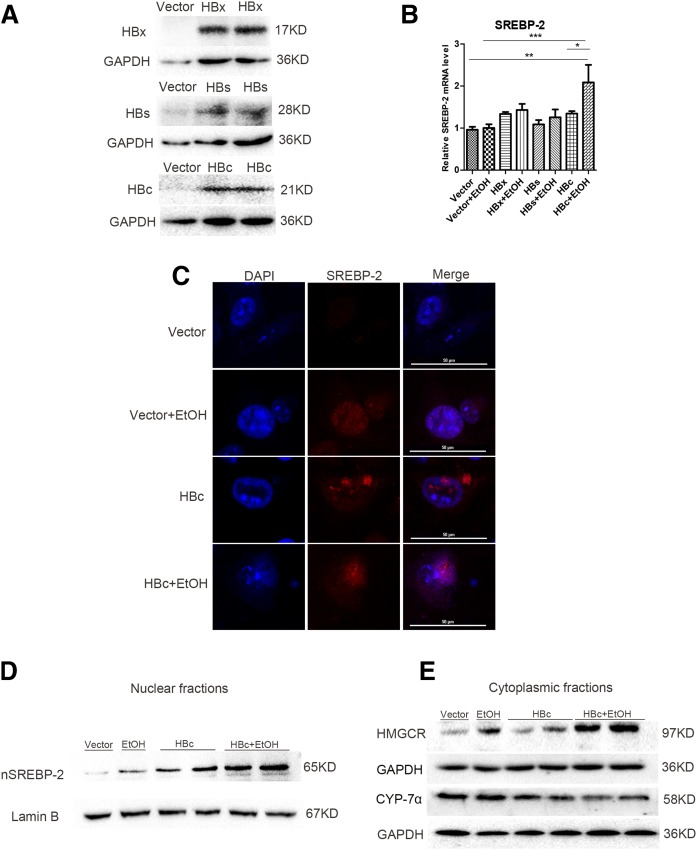
Overexpression of HBc protein combined with ethanol stimulation increases the cholesterol biosynthesis pathway and inhibits the cholesterol degradation pathway. A: The expression of HBx, HBs, and HBc in HepG2 cells after transfection with pCDH-HBx, pCDH-HBs, pCDH-HBc plasmid, or the empty vector. B: Gene expression analysis of SREBP-2 by real-time PCR in HepG2 cells. C: Expression of nuclear SREBP-2 (nSREBP-2) by immunofluorescence laser confocal microscopy in HepG2 cells. D: Western blots of nuclear SREBP-2 in HepG2 cells. E: Western blots of HMGCR and CYP-7α protein levels in HepG2 cells. Data are mean ± SEM of three separate experiments done in triplicate. *P < 0.05; **P < 0.01; ***P < 0.001.

To further understand whether HBV proteins directly affect the dysregulation of cholesterol metabolism and which HBV protein is involved, HBx-, HBs-, and HBc-expressing plasmids were created. The HBx, HBs, or HBc coding sequence was cloned into the pCDH-CMV-MCS-EF1-copGFP vector in-frame. The plasmid was transfected into HepG2 cells and the cell lysates were analyzed by Western blotting. As shown in **Fig. 6A**, specific protein bands were recognized by HBx-, HBs-, or HBc-specific antibody in cells transfected with pCDH-HBx, pCDH-HBs, or pCDH-HBc plasmid, but not in vector-transfected cells. The result demonstrated the successful expression of HBx, HBs, or HBc protein after transfection.

To explore the role of HBV proteins in cholesterol metabolism, we transfected HBx-, HBs-, HBc-expressing plasmid or empty vector into HepG2 cells with the ethanol treatment. Given the importance of SREBP-2 in modulating cholesterol metabolism, we first analyzed the SREBP-2 level. We found that overexpression of HBc protein with ethanol treatment significantly upregulated the mRNA level of SREBP-2, whereas HBx and HBs overexpression showed no marked upregulation on SREBP-2 level (Fig. 6B). Further, our experiment showed a significant increase of SREBP-2 nuclear translocation and protein expression in HBc overexpression combined with ethanol treatment (Fig. 6C, D). In this regard, our results showed HBc overexpression combined with ethanol treatment leads to further increase of HMGCR and decrease of CYP-7α in protein levels compared with other groups (Fig. 6E). These results indicated that HBc protein may directly activate the cholesterol biosynthesis pathway and inhibit degradation of cholesterol.

## DISCUSSION

An increasing number of ALD patients are infected with HBV, but few intensive studies have focused on the comorbidities, perhaps because of the absence of appropriate experimental models. A previous study has reported a HBV transgenic SCID mouse model treated with ethanol (39), in which ethanol consumption promoted HBV replication. Recently, an increasing number of reports have found that the body’s immune function and the lipid metabolism influence each other (40, 41). It can be suggested that the mouse model with complete immunity is a useful supplement to study lipid metabolism.

As HBV genotype B is one of the most common genotypes in the Chinese population, we chose the HBV replicons containing a replication-competent 1.3-fold over-length HBV genome from genotype B HBV. With the heterogeneity of host antiviral immunity, the immunocompetent mice in different strains of HBV genotype B may display different courses of disease. C57BL/6 and Balb/C mice showed an acute viral clearance following exposure to HBV genotype B by hydrodynamic injection; however, FVB/N mice developed intracellular HBV persistence and subsequently provided a convenient means to explore the host and viral factors associated with HBV persistence in vivo (32). Therefore, to further study the impacts of HBV combined with alcohol exposure on cholesterol metabolism, we constructed a murine-immunocompetent model of hepatic HBV persistence with alcoholic fatty liver via hydrodynamic injection of a pGEM-4Z1.3HBV vector and feeding an ethanol diet using FVB/N mice. In this model, characteristic hepatic HBV persistence and alcoholic fatty liver were present. HBeAg was persistently detected in the serum and HBcAg was positively expressed in the liver. After 6 weeks on the ethanol diet, mice exhibited fatty liver pathology with mild inflammation and increased cholesterol levels in the serum. This model may partly mimic the CHB patient with ALD.

The liver plays a central role in cholesterol homeostasis. The current study provides evidence for multiple and complex alterations in the pathways of cholesterol homeostasis in response to HBV infection (11, 12). Previously, whether ethanol consumption combined with HBV had a synergistic effect on cholesterol metabolism was poorly investigated. In our study, we observed that the total cholesterol levels in plasma and liver were both elevated significantly in alcohol consumption combined with HBV-persistence mice. This demonstrated that the combination of ethanol and HBV resulted in hepatic cholesterol accumulation. This accumulation may be through a combination of increased production and decreased degradation of cholesterol.

The biosynthesis of cholesterol involves the activation of numerous enzymes. In addition, SREBP-2 is a major transcription factor regulating the expression of many enzymes involved in this pathway (42–44). In general, SREBP-2 localizes to the endoplasmic reticulum. When activated, it is transported to the Golgi apparatus in the company with the SREBP cleavage activation protein, and then translocated to the nucleus. Following translocation, SREBP-2 performs as a nuclear transcription factor inducing cholesterol synthesis genes, such as HMGCR, the rate-limiting enzyme for cholesterol de novo synthesis (45–47). In our study, we found that ethanol treatment combined with HBV significantly activated SREBP-2 in the liver and thus increased expression of HMGCR, indicating that ethanol treatment combined with HBV synergistically enhanced cholesterol biosynthesis. This may partially account for increased hepatic total cholesterol contents in ethanol treatment combined with HBV-persistence mice.

LDLR is responsible for clearing circulating LDLs in the serum that are a risk factor of cardiovascular disease. This clearance is mainly mediated by SREBP-2 (48). Prior studies demonstrated that LDL inhibited transcription of the LDLR gene via suppressing the SREBP-2 pathway (49). Interestingly, in our study, we observed a significant downregulation of LDLR and upregulation of PCSK9 accompanied by increased SREBP-2 in nucleus. This dysregulation may additionally have decreased the hepatic uptake of LDL resulting in the observed elevation of serum LDL level, even though SREBP-2 was activated. These results implied that, other than SREBP-2 activation, upregulation of PCSK9 may be involved in the observed changes in cholesterol uptake.

Biosynthesis of bile acids is an important utilization of cholesterol. As the rate-limiting enzyme for bile acid synthesis, CYP-7α is responsible for converting cholesterol into 7-hydroxycholesterol and, hence, into bile acids within hepatocytes (50). Because the liver is the only organ for cholesterol removal via bile acid synthesis, it is possible that ethanol treatment combined with HBV may affect bile acid synthesis. In our study, we demonstrated significantly decreased CYP-7α mRNA and protein levels in alcohol consumption combined with HBV persistence mice. This suggested that ethanol and HBV synergistically inhibited the hepatic cholesterol metabolism pathway, which may result in the accumulation of cholesterol in the liver.

In addition, the cholesterol uptake and degradation pathway may be influenced in different stages of HBV infection. A recent study demonstrated that modulating hepatic cholesterol uptake by ezetimibe inhibits early HBV infection, with no effect after the HBV viral genome transduction (51). Binding of HBV to NTCP limits its function, thus promoting compensatory bile acid synthesis and cholesterol provision simultaneously, with enhancement of genes involved in transcriptional regulation, biosynthesis, and uptake of cholesterol, including SREBP-2, HMGCR, LDLR, and CYP7α (12). Our observation on decreased LDLR and CYP-7α may have arisen because of different stages of HBV infection, as well as the alcoholic factor.

HBV has a partially double-stranded circular DNA genome coding for the X (HBx), surface, core proteins, and polymerase. Recent studies have shown that HBX activates SREBP-1, the other isoform of SREBP, through interaction with C/EBP or liver X receptor alpha, accounting for fatty acid synthesis (52–54). Interestingly, in our study, we found that only HBc, but not HBx or HBs, participates in cholesterol metabolism. HBc protein expression combined with ethanol treatment significantly upregulated the SREBP-2 and thus increased the HMGCR downstream. In addition, HBc protein expression combined with ethanol treatment inhibited CYP-7α, thus suppressing the degradation of cholesterol.

In summary, we have successfully constructed a novel mouse model of ALD involving ethanol consumption combined with HBV persistence. Using this model, we demonstrated that ethanol and HBV together synergistically enhance cholesterol biosynthesis and decrease cholesterol utilization and uptake in vivo and in vitro. These changes collectively resulted in dysregulated cholesterol homeostasis that may contribute to the progression of various coexisting diseases. HBc protein may be a potential mechanism that directly affects cholesterol metabolism.

## Supplementary Material

Supplemental Data
