# Supplementary material for: Intracellular hepatitis B virus increases hepatic cholesterol deposition in alcoholic fatty liver via hepatitis B core protein
Source: J Lipid Res. 2017 Nov 13;59(1):58–68. doi: 10.1194/jlr.M079533 (PMC5748497; doi:10.1194/jlr.M079533)
Supplement: Supplemental Data [file supp_59_1_58__index.html]

Intracellular hepatitis B virus increases hepatic cholesterol deposition in alcoholic fatty liver via hepatitis B core protein — Intracellular hepatitis B virus increases hepatic cholesterol deposition in alcoholic fatty liver via hepatitis B core protein — Supplemental Data 

# Intracellular hepatitis B virus increases hepatic cholesterol deposition in alcoholic fatty liver via hepatitis B core protein

## Supplemental Data

- Supplemental Table S1 (.pdf, 92 KB) - Primer for polymerase chain reaction of HBx, HBs and HBc genes.
- Supplemental Table S2 (.pdf, 103 KB) - Primer for real-time polymerase chain reaction.
- Supplemental Table S3 (.pdf, 97 KB) - Serum biomarkers of liver function.
- Supplemental Figure S1 (.pdf, 114 KB) - Alcohol exposure combined with HBV persistence influenced cholesterol metabolism pathway in Huh7 cells.
