# Supplementary material for: Intracellular hepatitis B virus increases hepatic cholesterol deposition in alcoholic fatty liver via hepatitis B core protein
Source: J Lipid Res. 2017 Nov 13;59(1):58–68. doi: 10.1194/jlr.M079533 (PMC5748497; doi:10.1194/jlr.M079533)
Supplement: Supplemental Data [file 10.1194_M079533_jlr.M079533-1.pdf]

Supplementary Materials

Supplemental Table S1.

Gene	Primer for polymerase chain reaction
HBx	Sense :5’-agaGAATTCaccATGGCTGCTAGGCTGTACTGCC-3’
	Antisense : 5’-TTCGCGGGCCGCTTAGGCAGAGGTGAAAAAGTTGC-3’
HBs	Sense : 5’-aatGAATTCaccATGGGAGGTTGGTCTTCCAA-3’
	Antisense : 5’-gggGGATCCTCA AATGTATACCCA AAG AC-3’
HBc	Sense : 5’-gatTCTAGAaccATGCAACTTTTTCACCTCTG-3’
	Antisense : 5’-aagGAATTCCTA ACATTGAGATTCCCGAG-3’

Supplemental Table S1.Primer for polymerase chain reaction of HBx, HBs and HBc genes.

HBx : hepatitis B X gene

HBs : hepatitis B surface gene

HBc : hepatitis B core gene
