# Supplementary material for: Intracellular hepatitis B virus increases hepatic cholesterol deposition in alcoholic fatty liver via hepatitis B core protein
Source: J Lipid Res. 2017 Nov 13;59(1):58–68. doi: 10.1194/jlr.M079533 (PMC5748497; doi:10.1194/jlr.M079533)
Supplement: Supplemental Data [file 10.1194_M079533_jlr.M079533-2.pdf]

Supplemental Table S2.

Gene	Primer for real-time polymerase chain reaction
mβ-actin	Sense: 5’-GGTCAGAAGGACTCCTATGTGG-3’
	Antisense:5’-TGTCGTCCCAGTTGGTAACA-3’
mHMGCR	Sense: 5’-GGTTCTTTCCGTGCTGTGTT-3’
	Antisense:5’-CCATTTTTAACCCACGGAGA-3’
mSREBP-2	Sense: 5’-ACAGACACAAGGGCTAGGCT-3’
	Antisense:5’-GTGCTTCAACCCCACCTACT-3’
mLDLR	Sense: 5’-GAACTCAGGGCCTCTGTCTG-3’
	Antisense:5’-GAAACCATGCGTGTATCCCT-3’
mCYP-7α	Sense: 5’-CAGGGAGATGCTCTGTGTTCA-3’
	Antisense:5’-AGGCATACATCCCTTCCGTGA-3’
hHMGCR	Sense: 5’-TGATTGACCTTTCCAGAGCAAG-3’
	Antisense :5’-CTAAAATTGCCATTCCACGAGC-3’
hSREBP-2	Sense : 5’-CGTCCACCACCGACAGATGA-3’
	Antisense:5’-GAAGGCTGGAGACCAGGAAGA-3’
hGAPDH	Sense: 5’- GGTCGGAGTCAACGGATTTG-3’
	Antisense: 5’ GGAAGATGGTGATGGGATTTC-3’

Supplemental Table S2.Primer for real-time polymerase chain reaction.

HMGCR:3-hydroxy-3-methylglutaryl Coenzyme A reductase

SREBP-2:sterol regulatory element-binding protein 2

LDLR:low density lipoprotein receptor

CYP-7α:cholesterol 7 alpha-hydroxylase
