# Supplementary material for: Intracellular hepatitis B virus increases hepatic cholesterol deposition in alcoholic fatty liver via hepatitis B core protein
Source: J Lipid Res. 2017 Nov 13;59(1):58–68. doi: 10.1194/jlr.M079533 (PMC5748497; doi:10.1194/jlr.M079533)
Supplement: Supplemental Data [file 10.1194_M079533_jlr.M079533-3.pdf]

Supplemental Table S3

Biomarkers	Pair-fed(n=7)	EtOH(n=9)	HBV+Pair-fed(n=7)	HBV+EtOH(n=8)
TBIL(μmol/L)	<0.7	1.54±0.46	<0.7	1.26±0.17
DBIL(μmol/L)	<0.7	0.92±0.16	<0.7	0.91±0.08
ALP(U/L)	90.43±7.44	72.00±22.75	90.38±9.41	91.64±13.37
γ-GT(U/L)	<3	<3	<3	<3

Supplemental Table S3. Serum biomarkers of liver function.

TBLI :total bilirubin

DBIL :direct bilirubin

ALP:alkaline phosphatase

γ-GT:γ-glutathione
