# Supplementary material for: Intracellular hepatitis B virus increases hepatic cholesterol deposition in alcoholic fatty liver via hepatitis B core protein
Source: J Lipid Res. 2017 Nov 13;59(1):58–68. doi: 10.1194/jlr.M079533 (PMC5748497; doi:10.1194/jlr.M079533)
Supplement: Supplemental Data [file 10.1194_M079533_jlr.M079533-4.pdf]

Supplemental Figure S1

A

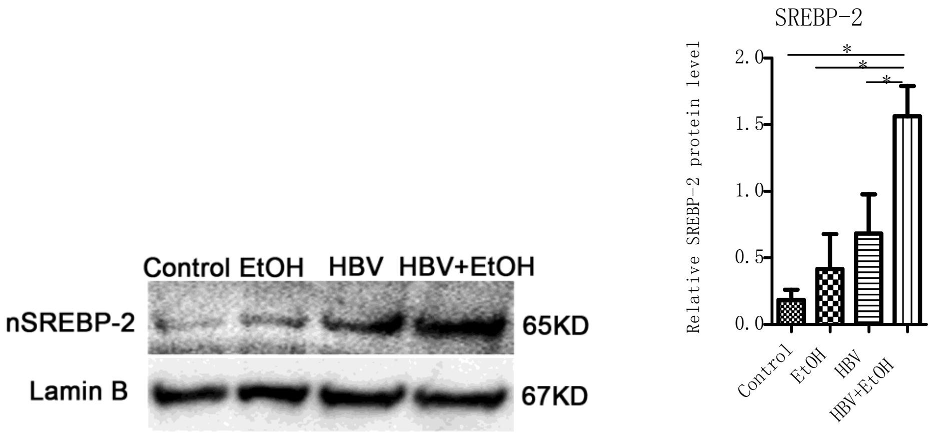

B

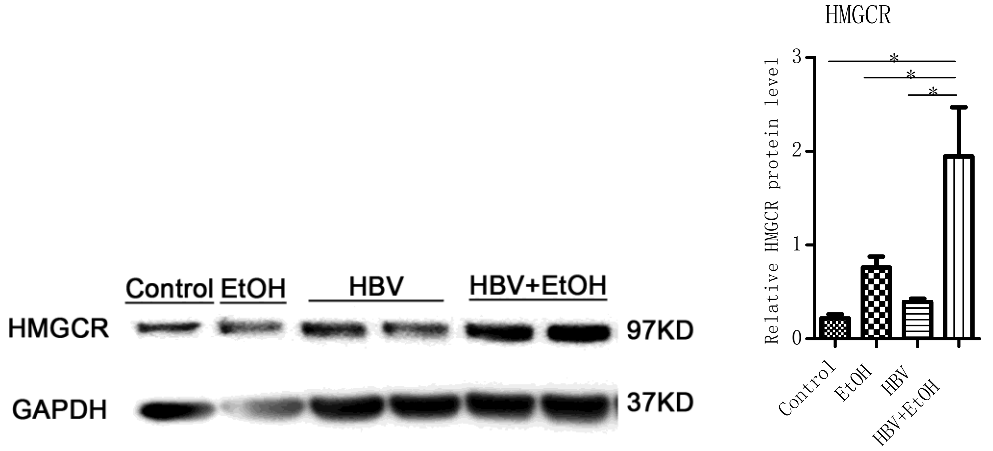

C

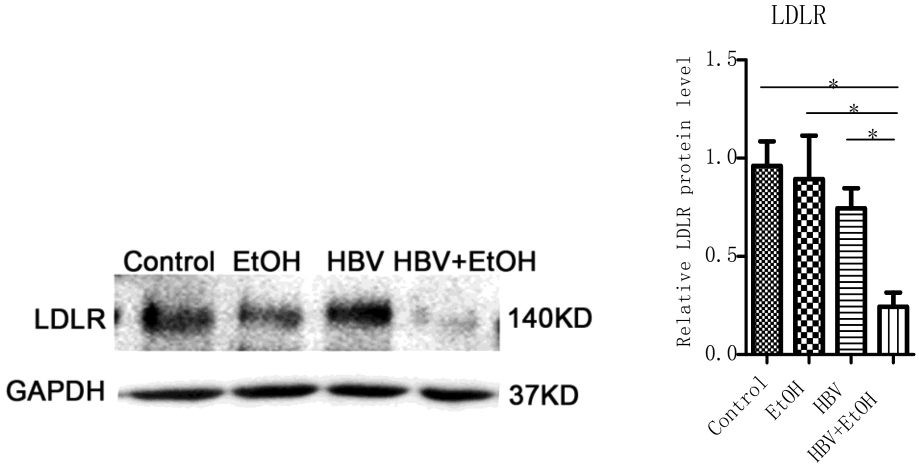

Supplemental Fig.S1 Alcohol exposure combined with HBV persistence influenced cholesterol metabolism pathway in Huh7 cells. Huh7 cells were transfected with pGEM-4Z1.3HBV vector or pGEM-4Z control vector, after 24h of transfection, cells were exposed to ethanol at concentration of 100mM or PBS for another 24h, then cells were harvested for further analysis. A. Representative Western blots and relative quantitation of nuclear SREBP-2 protein levels in Huh7 cells(n=3). Lamin B was used as a control loading. B. Representative Western blots and relative quantitation of HMGCR protein levels in Huh7 cells(n=3). C. Representative Western blots and relative quantitation of LDLR protein levels in Huh7 cells(n=3). GAPDH was used as a control loading. Data are means ±SEM.*P <0.05.
